# Sepsis modelling: current approaches and organ-on-chip perspectives

**DOI:** 10.1016/j.ebiom.2026.106120

**Published:** 2026-02-14

**Authors:** Mariana J. Silva, Gustavo W. Fehrenbach, Robert Pogue, Patrick Murray, Emanuele Rezoagli, John G. Laffey, Emma J. Murphy

**Affiliations:** a Bioengineering (Organ-on-Chip) Research Group, Centre for Applied Bioscience Research, Moylish Campus, Technological University of the Shannon, Limerick, Co. Limerick, V94 EC5T, Ireland; b PRISM Research Institute, Department of Engineering, Athlone Campus, Technological University of the Shannon, Athlone, Co. Westmeath, N37 HD68, Ireland; c Programa de Ciências Genômicas e Biotecnologia, Universidade Católica de Brasília, 71966-700, Brasilia, Brazil; d Programa de Biotecnologia, Universidade Católica Dom Bosco, 79117-900, Campo Grande, Brazil.; e LIFE Research Institute, Moylish Campus, Technological University of the Shannon, Limerick, Co. Limerick, V94 EC5T, Ireland; f School of Medicine and Surgery, University of Milano-Bicocca, Fondazione IRCCS San Gerardo dei Tintori, Monza, Italy; g Anaesthesia and Intensive Care Medicine, School of Medicine, Regenerative Medicine Institute at CÚRAM Research Ireland Centre for Medical Devices, University of Galway, H91 TK33, Ireland

**Keywords:** Sepsis, Experimental models, Translational relevance, Microfluidic systems, Organ-on-chip

## Abstract

Sepsis is a complex life-threatening condition involving immune dysregulation, endothelial dysfunction, and multi-organ failure. To investigate molecular and systemic processes driving disease progression, *in vitro*, *in vivo*, and *ex vivo* experimental methods have been developed. While these systems have advanced understanding of immune activation, cytokine signalling, and organ injury, differences in complexity, reproducibility, and alignment with human pathophysiology have limited the translation of many promising preclinical findings into clinical success. This review examines current literature on sepsis systems, evaluating them in terms of biological complexity, reproducibility, ethical constraints, and clinical applicability. In parallel, it discusses the potential use of microfluidic technology, particularly organ-on-chip, in replicating human physiology and capturing key features of sepsis. By comparing conventional and advanced systems, this review outlines challenges in sepsis research and identifies key directions for a more integrated approach to sepsis modelling, aiming to improve translational outcomes and therapeutic discovery. We aim to provide a structured basis for comparing models when selecting approaches for a given question or candidate therapy.

## Introduction

**Fig. 1:**
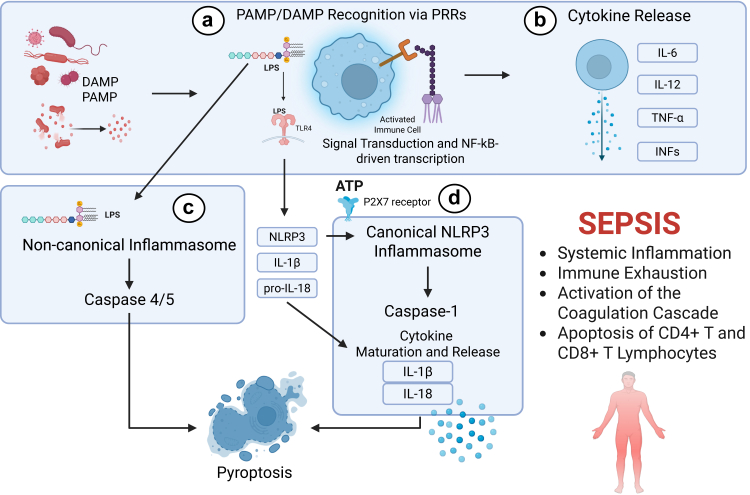
**Molecular mechanisms underlying sepsis pathogenesis.** (a) Pathogen-associated molecular patterns (PAMPs) and damage-associated molecular patterns (DAMPs) are recognised by pattern-recognition receptors (PRRs), such as the Toll-like receptor 4 (TLR4) on immune cells. This triggers intracellular signalling cascades and transcriptional activation via nuclear factor kappa-light-chain-enhancer of activated B cells (NF-kB); (b) Pro-inflammatory cytokines (IL-6, IL-12, TNF-α) and interferons (IFNs) are released contributing to systemic inflammation; (c) Cytosolic detection of intracellular lipopolysaccharide (LPS) activates the non-canonical inflammasome via caspase-4/5, promoting pyroptosis and further immune activation; (d) The canonical NLRP3 inflammasome is activated by lipopolysaccharide (LPS) priming and a secondary stimulus such as extracellular adenosine triphosphate (ATP) via the P2X7 receptor. This leads to caspase-1 activation, cleavage of pro-IL-1β, and pro-IL-18, secretion of these mature cytokines, and pyroptosis. Together, these processes contribute to systemic inflammation, immune exhaustion, coagulation cascade activation, and lymphocyte apoptosis, all features of sepsis. Created in BioRender. Juca, M. (2026) https://BioRender.com/pz8oezs.

Sepsis remains a major global health challenge, accounting for around 48.9 million cases and 11 million deaths annually.^1^^,^^2^ It is defined as an organ dysfunction caused by a dysregulated host response to infection, involving both pro-inflammatory and anti-inflammatory pathways, along with other non-immunological dysfunctions. The activation of these pathways leads to an exacerbated release of cytokines and mediators, resulting in the initiation of coagulation and complement cascades (Fig. 1).^3^ Sepsis can lead to impaired function of several organs, including the lungs (ARDS), kidneys (AKI), brain (confusion, delirium, or coma), liver, and cardiovascular system (shock or myocardial dysfunction).^4^

Preclinical models have significantly contributed to the understanding of sepsis pathophysiology. They include *in vitro*, *in vivo*, *ex vivo*, and *in silico* approaches. All these models have been fundamental for testing therapeutic potentials and understanding inflammatory cascades, endothelial injury, and organ dysfunction in sepsis. As in many fields, progression from experimental findings to patient benefit remains limited.^4^, ^5^, ^6^, ^7^, ^8^ NIH reports that 80–90% of therapeutic candidates for sepsis never reach human testing, and fewer than one in a thousand achieves FDA approval.^9^^,^^10^ Low translation is not unique to sepsis. From 2015 to 2023 infectious disease programs recorded a 2.6% success rate, rising to 4.4% when COVID 19 trials are excluded, with 34.7% success at phase 3.^11^

**Fig. 2:**
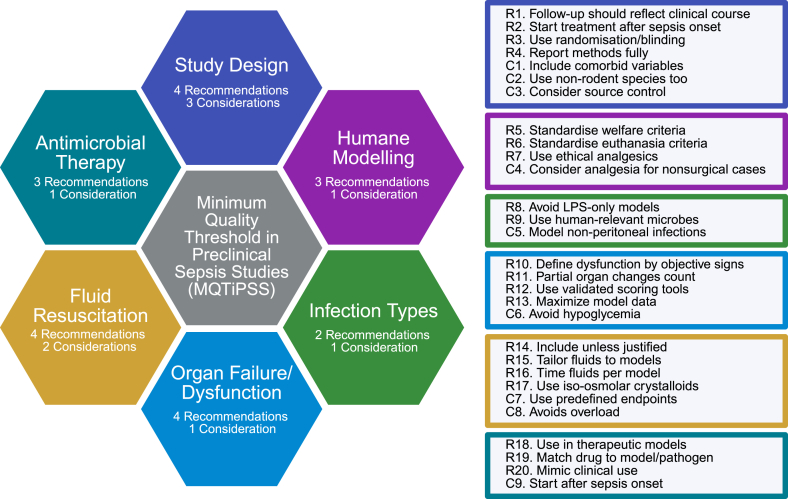
**Working groups and associated recommendations (R) and considerations (C) established by the MQTiPSS framework.** Each coloured hexagon represents one working group: Study Design, Humane Modelling, Infection Types, Organ Failure/dysfunction, Fluid Resuscitation, and Antimicrobial Therapy. Boxes summarise the key recommendations and considerations in each domain, addressing aspects of experimental standardisation, reproducibility, ethical practice, and translational relevance in animal sepsis models. Created in BioRender. Juca, M. (2026) https://BioRender.com/wfjvtwv.

The publication of the Minimum Quality Threshold in Pre-Clinical Sepsis Studies (MQTiPSS) in 2018 marked a critical step towards improving the quality and reliability of sepsis animal models. It introduced 29 consensus criteria across 6 domains including Study Design, Humane Modelling, Infection types, Organ Failure/dysfunction, Fluid Resuscitation, and Antimicrobial Therapy.^12^^,^^13^ The MQTiPSS aims to improve translational value of preclinical animal studies by suggesting strategies to advance the quality of sepsis models (Fig. 2).

Despite progress, there is still a gap between preclinical findings and patient outcomes. Several advanced models are now being explored to address more complex questions, including organoids, *ex vivo* human tissues, computational approaches, and microfluidic organ-on-chip devices. These systems can capture features such as immune cell movement and time-resolved responses to injury, which may help identify mechanisms of inflammation and organ dysfunction in sepsis. Evidence is still developing and comparative studies are needed.^6^^,^^14^, ^15^, ^16^

Sepsis modelling has come a long way, and new models are advancing quickly. This work provides a summary of existing literature on established sepsis models, exploring both their applications and limitations. *In vitro*, *in vivo*, *in silico*, and *ex vivo* methods are discussed in terms of their practical value, physiological relevance, reproducibility, standardisation, and ethical implications. Lastly, future directions for sepsis research are examined, including potential models using microfluidics.

## *In vivo* models

Animal models are employed to simulate the complexity of host-pathogen interactions. Among them, mouse models are often used because of their relative low cost, short generation time, ease of experimentation, and availability of genetically identical or genetically modified strains.^17^ They are considered reproducible systems for studying the pathogenesis of sepsis and demonstrate potential in preliminary testing of therapeutic agents. Other small animals such as rats, hamsters, rabbits, and dogs have also been used but are not largely explored.^18^ Large mammals such as pigs, sheep, and non-human primates are often used to study conditions related to clinical intensive care settings.^19^ Here, we summarise different murine models of sepsis, focusing on their methods and limitations.

### *In vivo* PAMP/DAMP-induced sepsis models

The use of PAMPs and DAMPs imitate induction of an inflammatory reaction. They may be administered through intravenous, intraperitoneal, or intranasal/intratracheal processes, in which an immediate innate-immune inflammatory response is induced. *In vivo* endotoxin administration is typically utilised to study the instant biology of septic shock, where there is a high Toll-like receptor signalling.^19^ These models have made possible the clarification on roles of inflammatory pathways and cytokines, critical for sepsis.^7^

Although one should not overstate their influence, in comparison to humans, animals are generally less sensitive to endotoxins, as they require a higher dose to display symptoms.^14^^,^^20^ Additionally, human and mouse responses vary in cytokine kinetics and time of death, potentially due to differences in expression of protective proteins such as haemopexin.^17^ Part II of the MQTiPSS for Types of Infections and Organ Dysfunction Endpoints, illustrated in Fig. 2, states that one of the defining elements for sepsis is the presence of an infection. Therefore, it recommends that infectious insults using bacteria should be used over bacterial components, such as PAMPs and DAMPs.^12^

### Live pathogen injection and two-hit models

Bacterial injection models, involving the administration of live pathogens via intra-peritoneal, intravenous, or intratracheal routes, have been long used to mimic a real-life sepsis inflammation-infection scenario in mice. *Escherichia coli*, *Staphylococcus*, and *Pseudomonas* are often administered as pure or mixed cultures.^7^ These models help study host-pathogen responses but require high bacterial load.^17^ Response also depends on strain, dosage, and host susceptibility, and if not standardised, these injections may cause intoxication rather than true infection.^7^^,^^20^

A variation of this model, known as the “two-hit” model, allows the investigation of sepsis beyond the abdominal cavity. This approach involves, for example, the initial induction of abdominal sepsis *in vivo*, followed by a secondary administration of bacteria to a target organ. Bacteria may be introduced via the respiratory tract to simulate hospital-acquired pneumonia in patients following trauma or abdominal sepsis. This technique can also be used to reproduce bacterial injection into the bladder to model ascending urinary tract infections.^7^^,^^17^

### Intraperitoneal polymicrobial sepsis models: CLP, CS, and CASP

Intraperitoneal models are widely regarded as the standard for mimicking sepsis *in vivo*. The three most used methodologies are called caecal ligation and puncture (CLP), caecal slurry (CS), and colon ascendens stent peritonitis (CASP).^7^

CLP is referred as the gold standard for capturing the main features of immune response in polymicrobial abdominal sepsis, comprising around 44% of all sepsis animal studies.^12^ This method involves puncturing the caecum, applying gentle pressure to extrude a small amount of its contents, and subsequently repositioning it into the abdominal cavity, followed by closure of the abdomen using running sutures. An advantage of CLP lies in its ability to modulate the severity of sepsis through the controlled adjustment of parameters such as needle size, number of caecal punctures, and the use of antibiotics.^14^^,^^21^ CLP is well researched and widely characterised. However, it's associated with substantial variation in outcomes, even in standardised conditions.^19^ CASP consists of a leakage of the colonic contents into the abdominal cavity. While this leakage induces an exacerbated inflammation, it lacks precise information on microbial composition, decreasing relevance in the model.^22^ Caecal slurry (CS) injection is based on intra-peritoneal injection of caecal slurry from one or multiple donors, which has been standardised.^23^ Its biggest advantage lies in the fact that it can be performed in neonatal mice, in order to study effects of sepsis in human neonates. However, challenges lie in reproducibility of the experimental design, as each experiment determines the dose required to induce sepsis over a certain period.^17^ Studies have introduced reproducible methods using standardised polymicrobial populations from mice and/or human stool expanded in media that could be long-term stored for a future use in glycerol. This variation aims to decrease variability, as well as introduce human-derived samples.^23^, ^22^, ^24^

It remains challenging to mimic the downstream pathological characteristics of severe clinical sepsis and maintain a high survival rate in *in vivo* models. A recent animal study similarly identified the moment of physiologic decompensation in real time to guide testing of candidate treatments.^25^ To mimic clinical care, infectious models often include antibiotics, yet intervention timing varies widely. Most studies give antibiotics immediately or within a few hours of the infectious insult. Because sepsis progresses faster in animals, early antibiotics may be reasonable in models, but patients are rarely treated within such a narrow window.^26^ In a severe caecal slurry mouse model, early antibiotics given at 1–6 h rescued most animals but reduced bacteraemia, cytokinaemia, and organ injury. Delaying antibiotics alone to 12–24 h or later caused high mortality. Starting antibiotics plus fluid resuscitation at 12 h or later, after bacteraemia had developed, produced about 75% survival, falling to 50% at 24 h, with bacteraemia, elevated cytokines, organ injury, and prolonged weight loss, indicating a longer disease course that aligns with ICU phase pathology.^26^

In a study of severe abdominal infection with caecal slurry injection in 17–18-week C57BL/6 mice, early antibiotic and imipenem at 1 or 6 h rescued most animals but produced little bacteraemia, cytokinaemia, or organ injury. Delaying antibiotics to 12 or 24 h led to high mortality, but adding subcutaneous fluids with delayed antibiotics yielded about 75% survival, with bacteraemia, elevated cytokines, organ dysfunction, and prolonged weight loss below 90% for 4 weeks. The study recommends starting antibiotics plus fluids at a late time point after bacteraemia to mimic pathology while maintaining survival for study.^26^ It achieves an ICU phase trajectory with sufficient survival for study.

*In vivo* models can show reduced endotoxin sensitivity, differences in cytokine kinetics, and time of death, dependence on strain, dose, and host susceptibility, risk of intoxication rather than infection, variation in outcomes, imprecise microbial composition, and challenges in reproducibility and dosing. They also offer low cost, short generation time, ease of experimentation, availability of genetically identical or modified strains, use in studying sepsis pathogenesis, adjustable severity, and applicability to neonatal sepsis and secondary infections.

## *In vitro* models

*In vitro* models are key tools for studying cellular and molecular mechanisms in inflammation.^27^ Through controlled manipulation of isolated cells, traditional cultures reveal responses to infectious or inflammatory stimuli. Common cell types include THP-1 macrophages and human umbilical vein endothelial cells (HUVECs), exposed to PAMPs, DAMPs, or cytokines, to induce sepsis-like inflammation. These models are simplified, reproducible, and are capable of dissecting signalling pathways related to inflammation. They are highly used as a cheaper option in preliminary studies to screen effects of potential therapeutic agents, where later on they can be tested in more complex systems.^14^

### LPS-based *in vitro* sepsis models

Among the most widely used stimuli for *in vitro* sepsis modelling is the administration of lipopolysaccharide (LPS) to cultured cells, either by adding it to the cell culture medium or by transfecting directly into the cytosol. LPS is a PAMP derived from the cell wall of Gram-negative bacteria, and although it doesn’t cause an active infection, it can be used to obtain information on the main inflammatory pathways in sepsis.^14^ Extracellular LPS engages the TLR4-MD-2-CD14 complex, activating canonical NF-κB and IRF3 pathways, and triggering the release of pro-inflammatory cytokines such as IL-1β, IL-6, TNF-α, and MCP-1.^28^ In contrast, intracellular LPS initiates non-canonical inflammasome activation via caspase-4/5 activation, causing cleavage of gasdermin D, inducing pyroptosis, and potentially triggering secondary inflammasome activation.^26^

Gram-negative bacteria are the most common cause of sepsis infection.^29^ Their outer-membrane LPS has three parts lipid A, core sugars, and O antigen with lipid A inducing most bioactivity. LPS is not a single molecule but a heterogeneous class whose composition varies by species and conditions, forming different supramolecular aggregates on release this heterogeneity affects any modelling approach.^30^

LPS *in vitro* does not create active infection and depends on artificial stimulation.^14^ *In vivo*, LPS binds LBP, but LBP is absent *in vitro* unless added, reducing sensitivity.^28^ Models range from single-hit LPS assays to two-stimulus and double-hit designs, with wide variation in cell type, dose, and LPS quality. Lot-to-lot differences occur, with some new *E. coli* endotoxin lots stronger or weaker than older lots that retained potency for ten years. Dosing method and assay limits also influence outcomes.^31^

Chen et al. showed that 100 ng/mL LPS induces strong responses in HUVECs, whereas other studies use higher doses, such as 1 μg/mL in THP-1 cells with a second PAMP stimulus.^32^^,^^33^ Dose and delivery determine response magnitude, and standardised preparations give reproducible effects without proportional increases at slightly higher bolus doses.^34^ Activity depends on aggregates rather than monomers, mixed aggregates with under-acylated lipid A or phospholipids increase TNF-α, while monomers show minimal activity.^35^ Human endotoxaemia uses purified GMP-grade LPS with dose-dependent TLR4-mediated responses.^36^ Intravenous models use 0.06–4 ng/kg (2 ng/kg validated), whereas respiratory and nasal studies use micrograms or endotoxin units (EU), and commercial variation supports standardisation.^37^ Earlier protocols used 3–5 ng/kg; current protocols often use 0.2–2 ng/kg.^37^ All these studies demonstrate that LPS quantity and quality strongly shape inflammatory outcomes.

### *In vitro* models with Gram-positive bacteria and mixed PAMPs

Alternative *in vitro* approaches have been incorporated using multiple PAMPs, such as LPS combined with peptidoglycan (PepG) or lipoteichoic acid, which are components of the Gram-positive bacterial cell wall.^38^ The stimulation of Gram-positive bacterial PAMPs has been shown to activate NOD-like receptors, which are located in the cytosol, producing a more complex profile that better reflects the heterogenicity in human sepsis. These strategies are used to increase specificity of the model, where the crosswalk between TLR and NOD-like receptors is crucial to produce an efficient innate immune response to pathogens.^14^

Many studies have unravelled LPS-induced sepsis *in vitro* along with other PAMPs. More specifically, Bird et al. induced sepsis-like inflammation by incubating HUVECs in 100 ng/mL of LPS and 100 ng/mL of PepG. In this study, the combination of stimuli led to surface expression of functional NOP receptors and downstream ERK1/2 phosphorylation, suggesting that LPS plus PepG triggered receptor translation, membrane trafficking, and activation of intracellular signalling pathways.^39^ Additionally, Sun et al. showed non-canonical NLRP3 inflammasome activation by combining intracellular LPS in primary BMDMs with Pam3CSK4, a TLR2/TLR1 ligand. This stimulation induced pyroptosis and promoted significant IL-1β secretion, confirmed by caspase-1 cleavage and western blotting analysis.^40^

### *In vitro* inflammation models triggered by DAMPs

DAMPs are endogenous molecules released from dying cells that contribute to debris clearance and tissue repair. They also serve as potent immune activators and are often used to simulate inflammation, both individually and in combination to PAMPs.^41^ Identified by many immune pattern recognising receptors, key DAMPs include: HMGB1, extracellular CIRP (eCIRP), ATP, NAD+, and nucleic acid-associated proteins.^33^ Murao et al. induced sepsis-like inflammation in murine peritoneal macrophages by treating the cells with eCIRP, validated by an increase in IL-6 and TNF-α levels, along with p38 phosphorylation and IκBα degradation.^42^

### Advanced *In vitro* models: Co-cultures, 3D cultures, and organoids

In response to limitations of traditional *in vitro* models, more representative models have been developed to study sepsis-related inflammation.^43^ Cell–cell interactions play a role in sepsis but are often absent in conventional monocultures.^44^ For example, the interactions between endothelial cells (ECs) and smooth muscle cells support blood vessel structure and function.^45^ In response, co-culture systems are used to better model the cell–cell interactions that underlie inflammation.^46^ Huang et al. demonstrated that exposure of macrophages and T-cells to LPS results in suppression of T-cell function by septic macrophages, contributing to sepsis-induced immunosuppression.^47^

While co-cultures improve the biological relevance of cell signalling and interactions, three-dimensional (3D) cell cultures offer a more relevant representation of the cellular microenvironment in comparison to traditional or co-cultured monolayer cells.^43^ They have enhanced cell–cell adhesion, improved functionality, and a more representative gene expression profile. For example, Liu et al. showed enhanced cytokine responses and LPS sensitivity in 3D-cultured A549 lung epithelial cells and HepG2 hepatocytes, compared to 2D cultures.^46^

Building on the structural and functional advantage of 3D cultures, organoids represent a further advancement of *in vitro* sepsis modelling. Organoids are 3D cell cultures derived from stem-cells that self-organise to recapitulate the main characteristics of organ function and structure. The stem-cells are embedded within an extracellular matrix with specific growth factors, enabling the formation of complex structures that contain multiple differentiated cell types.^48^ Huang et al. compared LPS-induced intestinal injury in a mice model and in intestinal organoids, demonstrating that the *in vitro* system closely replicates the *in vivo* biological responses associated with sepsis, providing organ-specific responses.^47^

Li et al. built a human iPSC derived liver organoid that includes resident Kupffer-like macrophages for modelling liver dysfunction in sepsis. They generated erythro myeloid progenitors from the same iPSC line, incorporated them into self-assembled liver organoids containing hepatic endoderm, endothelium, and mesenchyme, and used macrophage Colony Stimulating Factor to support retention and maturation. The organoids showed liver functions such as albumin secretion, CYP activity, ammonia clearance, Indocyanine green (ICG) handling, endothelial networks, and Kupffer cell marker expression with transcriptomes close to primary Kupffer cells. LPS plus IFN-γ triggered Kupffer activation, increased TNF-a and IL-6 higher ROS and cell death, loss of MRP2 signal and endothelial cells, and a drop in hepatocyte functions. Removing the stimulus enabled partial recovery**,** early TLR4 inhibition with TAK 242 reduced inflammation**,** and improved function in a dose dependent manner.^49^

In another study, authors built a microfluidic human liver-sinusoid organoid with vascular and hepatocyte layers plus non-parenchymal cells. TLR agonists triggered cytokine release and reduced VE-cadherin, MRP2, and ApoB, causing barrier loss and hepatocyte dysfunction. When added, human monocytes dampened inflammation and restored transporter activity and liver outputs. These advancements represent significant efforts towards human-relevant models of sepsis. Although organoids resemble structures of organs and are highly useful for disease research, they lack tissue–tissue interfaces and organ-level structures.^44^ Additionally, the variability of cellular aggregates formation and complex cell culture techniques present practical limitations.^43^

These *in vitro* approaches are simplified, reproducible, low-cost tools that enable controlled manipulation of isolated cells, pathway analysis, preliminary therapeutic screening, co-culture interaction studies, and organ-specific responses using 3D cultures and organoids. However, they rely on artificial stimuli rather than active infection, lack serum factors such as LBP, vary widely by cell line, quantity, and quality of LPS, may produce endotoxin tolerance, and often require higher LPS doses than needed physiologically. Additional limitations include dependence on multiple PAMP combinations, variability in cellular aggregate formation, absence of tissue–tissue interfaces and organ-level structures in organoids, and complex cell culture techniques. As a result, *in vivo* techniques continue to serve as the main models for sepsis research, although they may bring challenges from an ethical perspective.^50^

## Human-based sepsis models

Human LPS models involve giving a low dose of LPS to healthy volunteers to trigger a short-term inflammatory response. This approach lets researchers study early inflammation in humans and assess potential treatments in a controlled and reproducible setting from a known starting point. Intravenous LPS induces cytokine release and leucocyte trafficking. The response scales with doses across 0.06–4 ng/kg. Dosing at 2–4 ng/kg will induce strong inflammation (2 ng/kg validated for Systemic Inflammatory Response Syndrome), while lower doses model chronic, low-grade inflammation.^37^ Following intravenous LPS, symptoms begin within one hour with headache and myalgia. Tachypnoea develops within 2–3 hours. Body temperature increases by 1–3 °C, peaking at 3–4 hours, accompanied by tachycardia around 90–100 bpm. Mean arterial pressure decreases slightly at around 3 hours. By 24 hours, temperature and blood pressure return to baseline, while heart rate remains mildly elevated.^51^, ^52^, ^53^, ^54^, ^55^

In a randomised, double-blind, placebo-controlled study with single ascending low doses IV LPS (0.5–2 ng/kg) in healthy volunteers, produced dose-dependent increases in temperature, heart rate, CRP, and circulating cytokines within hours, with only mild symptoms. When blood from these subjects was stimulated *ex vivo* with LPS, cytokine release of TNF-α, IL-1β, and IL-6 was markedly reduced at 6 h after the *in vivo* dose, indicating transient hyporesponsiveness that resolved by 12 h. In contrast, at lower *in vivo* doses, *ex vivo* IL-6 and IL-8 release increased, suggesting immune cell priming.^52^

Human *ex vivo* whole blood models have been used for years to investigate pathological responses of immune cells in a relevant environment.^56^ By stimulating fresh blood with LPS or live bacteria, these assays capture cytokine dynamics, cell surface marker expression, and innate immune activation in a controlled setting, preserving interactions between leukocytes, plasma proteins, and the complement system.^46^ These studies have provided important evidence in sepsis research. In 62 surgical ICU patients and 15 healthy controls, heparinised whole blood was incubated with LPS (10 ng/mL) for 3 h to measure *ex vivo* TNF-α and IL-6. In unstimulated blood, cytokines were minimal, while in stimulated blood levels varied. Patients with low cytokine capacity (IL-6 < 3000 pg/mL and TNF-α < 2100 pg/mL) had higher infection rates, longer ICU stays, about seven more ventilator days, and higher mortality. Stimulated TNF-α correlated more closely with mortality than IL-6, supporting *ex vivo* LPS assays for identifying high-risk patients.^57^

Human intravenous endotoxin studies show that prior LPS exposure induces selective endotoxin tolerance on *ex vivo* restimulation, with TNF-α, IL-1β, IL-6, and IL-10 reduced and IL-1 receptor antagonist increased, partly via early soluble plasma factors.^58^ Sex differences are evident, with men showing greater temperature rises and larger drops in mean arterial pressure despite similar leucocyte, cytokine, and cortisol profiles.^59^

Unlike homogeneous *in vitro*, organoid, or mouse models, these studies capture acute systemic inflammation and subsequent immunotolerance. Early evidence of immune hyporeactivity in sepsis came from peripheral blood lymphocytes. Wood et al. showed reduced IL-2 after PHA stimulation, and later studies reported impaired IFN-γ production in sepsis and severe injury.^60^^,^^61^

Monocyte responses have been widely examined in isolated cells and whole blood. In sepsis, monocytes show reduced LPS-stimulated release of TNF-α, IL-1α, IL-1β, IL-6, IL-10, and IL-12, but not interleukin-1 receptor antagonist (IL-1ra). Blunted cytokine production is also seen with other stimuli, including silica, staphylococcal enterotoxin B, and killed *Streptococcus pyogenes* and *Staphylococcus aureus*.^62^ Beyond cytokines, whole-blood assays have shown that heat-killed *Klebsiella pneumoniae* and *S. aureus* but not purified LPS induce large platelet leucocyte aggregates that adhere to TNFα-activated endothelium, an effect reduced by P-selectin blockade or angiopoietin-2 trapping.^63^

Human LPS models provide a controlled, reproducible inflammatory challenge but do not replicate critical illness. Most IV studies give a single morning bolus to healthy young men, yet responses differ by sex. Women show higher TNF-α, IL-6, CRP, cortisol, and a larger fall in mean arterial pressure. Cytokine release is also higher at night. The single-bolus design fails to capture the prolonged or repeated stimuli seen in critical illness*. Ex vivo* approaches allow the study of real human samples yet limitations exist such as possible interference with the anticoagulation used within the system and the lack of interactions with organs, limiting the ability to simulate systemic inflammation.

## *In silico* models

To complement these systems, *in silico* methods serve as a valuable tool. Computational methods, especially data mining and machine learning are useful to phenotype, predict, and classify sepsis. The integration and analysis of already collected data serves as a minimal-cost instrument capable of guiding therapy, enhancing early diagnosis, and optimising clinical management.^64^

Molecular dynamics simulations may also be adopted to predict modelling of therapeutic agents for sepsis. The 3D structure of proteins involved in sepsis can be retrieved from databases and used to dock the structure of the agent with these proteins, analysing their binding and stability over time.^64^ These models are used in studies that do not account for other variables in the body during sepsis. Nevertheless, it opens doors for future research regarding potentials therapeutic interventions for sepsis and may help the discovery of possible biomarkers.

## Microfluidic technology

Microfluidics enables precise, automated modelling of disease with minimal reagents and supports biomarker discovery and drug screening.^65^ It allows real-time functional analysis of cells and is relevant in sepsis, where immune responses evolve with coexisting hyperactivation and immunosuppression.^66^^,^^67^ Organ-on-chip platforms apply flow and tissue interfaces, enabling measurements not possible in static cultures, including defined shear, real-time barrier assessment, immune cell captures and transmigration, controlled gradients, time-structured dosing, serial sampling, and linked-tissue interaction.^68^, ^69^, ^70^, ^71^, ^72^

**Fig. 3:**
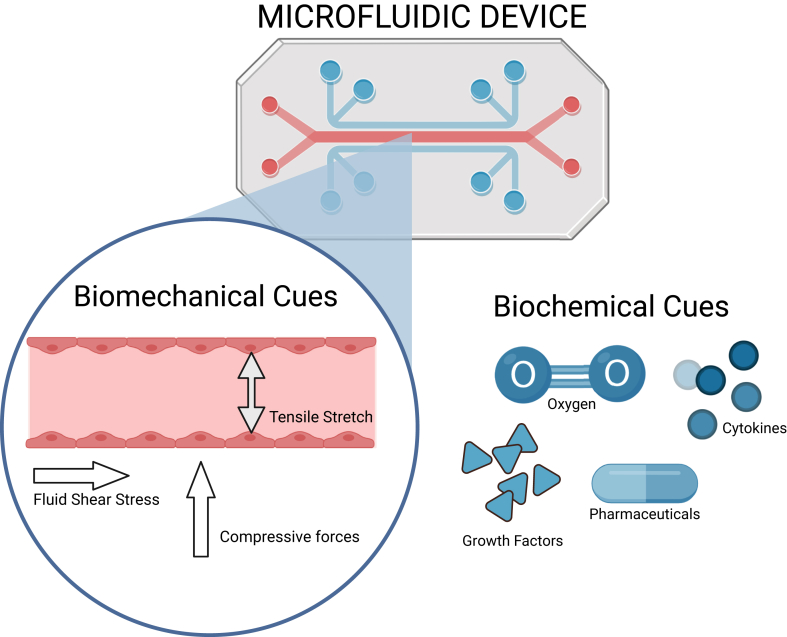
**Representation of biomechanical and biochemical cues within a microfluidic organ-on-chip device.** Microfluidic platforms support simultaneous application of stimuli such as oxygen gradients, cytokines, pharmaceuticals, and growth factors. Analysis of fluid shear stress, tensile stretch, and compressive forces can be done, allowing for the dynamic study of cellular responses under physiologically relevant conditions. Created in BioRender. Juca, M. (2026) https://BioRender.com/r9e3nob.

Sepsis’ immune coexistence affects mortality, secondary infections, and long-term complications. Macrophage M1/M2 balance influences sepsis progression and although CLP captures this transition, timing varies.^26^^,^^73^^,^^74^ Microfluidics can reproduce biomechanical and biochemical cues central to tissue function (Fig. 3).^75^ This is valuable in sepsis, where endothelial dysfunction alters shear forces and vascular tone, and organ-on-chip (OOC) systems can model these dynamics.^76^

Microfluidic models have addressed sepsis-related organ dysfunction. Zhu et al. built a co-culture device for neuroinflammatory signalling in sepsis-associated encephalopathy.^77^ Other chips exposed endothelial cells to sepsis-patient plasma, showing increased permeability, monolayer disruption, and higher ICAM-1 and IL-6.^78^ Multi-organ interactions can be studied using fluid-linked systems, and multiorgan-on-chip models may clarify sepsis-related organ damage.^79^ Although no system models full systemic crosstalk, linked chips have shown gut–liver PK interactions and automated platforms have maintained eight vascularised chip units with shared perfusion and tracer based transport.^80^ These demonstrate feasibility for multi-organ sepsis modelling.

Immune integration remains limited, though essential for sepsis modelling.^81^ Adding immune cells is recent, and models without them diverge from normal physiology.^82^ Tissue-resident cells activate endothelium to recruit circulating immune cells.^81^ PAMP–PRR signalling drives inflammation, and activated endothelium mediates capture, rolling, arrest, crawling, and transmigration.^83^ Disturbed flow also activates endothelium.^84^, ^85^, ^86^ Extravasation is modelled with transwells or flow chambers, though each has limitations.^82^^,^^87^^,^^88^

Most infection-on-chip models still lack full immune inclusion. Primary epithelial cells are scarce, so models use cell lines such as cancer-derived lung epithelium and endothelial subtype affects immune behaviour.^82^ Adaptive studies require autologous or HLA-matched cells. iPSC-derived immune cells assist but may be immature.^79^ Systemic immune responses depend on vasculature, and reproducing permeability, shear, and transmigration consistently is difficult. Chip materials, ECMs, hydrogels, mechanical forces, and measurement methods also influence immune behaviour.^79^

PDMS absorbs hydrophobic small molecules, reducing their effective concentration.^89^ Absorption varies with time, coatings, and cells; amphiphilic coatings reduce uptake. Computational–experimental methods can predict drug absorption and concentration profiles in PDMS chips.^90^^,^^91^ Chips are low-throughput and costly, requiring higher-throughput or automated approaches.^72^ Plastics also rapidly form conditioning films that promote bacterial attachment and biofilms, altering pathogen behaviour in infection-focused models.^92^

Microfluidic and organ-on-chip systems enable real-time cell analysis under controlled changes, capture immune shifts, recreate biomechanical/biochemical cues, probe vascular dysfunction and barrier control, model sepsis-related organ injury, and support fluid-connected multi-organ studies with serial sampling. Limitations include incomplete immune integration, reliance on cell lines and scarce primary epithelia, use of venous rather than microvascular endothelium, challenges reproducing vascular permeability/transmigration, sensitivity to chip design and biomaterials, PDMS absorption of hydrophobic drugs, lower throughput and higher cost than conventional cultures, and plastic surface conditioning that alters pathogen behaviour.

## Comparative overview of sepsis experimental models

**Fig. 4:**
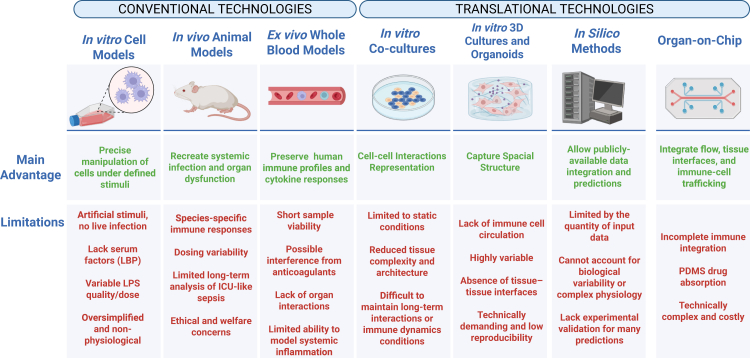
**Progression of preclinical sepsis models and emergence of translational technologies.** Conventional approaches, including *in vitro*, *in vivo*, and *ex vivo* models have supported mechanistic understanding but represent limitations in clinical translation. Advances in co-culture systems, 3D cultures, organoids, and *in silico* methods offer more complex and human-relevant platforms for studying systemic inflammation and sepsis. Created in BioRender. Juca, M. (2026) https://BioRender.com/o77hiwl.

As sepsis research advances, various experimental models have arisen, each designed to replicate different factors of this multifaceted disease. Given its complexity, no single model can fully replicate sepsis’ diverse pathophysiological spectrum. Instead, each platform offers specific advantages and limitations, with the choice of a singular model, or a combination, decided by the research question to be addressed. *In vitro* systems offer high experimental control over cellular pathways, whereas *in vivo* animal models provide insights into the host response. *Ex vivo* approaches utilise human samples for translational relevance, while organ-on-chip technologies aims to integrate human cells to mimic physiological contexts not yet fully explored in other systems (Fig. 4). Each model contributes uniquely to advance understanding of sepsis, while also presenting specific limitations in terms of complexity, reproducibility, ethics, and translational applicability. Mapping strengths and limits across models supports informed model choice. Table 1 summarises key insights from conventional and organ-on-chip models, listing primary readouts, experimental focus, and the biological information each yields.

**Table 1:** Comparative analysis of approaches for modelling sepsis.

Study	Model	Experimental focus	Cells/Samples examples	Measured outcomes	Reference
*In vivo*	Endotoxaemia Models	• Acute inflammation • TLR pathways • Organ-specific failure (e.g., lung, intestine) • Response to resuscitation	• Generally, mice (e.g., C57BL/6, BALB/c) or porcine models	• Cytokine and chemokine release • Pathway activations • Hypothermia • Survival rates	^17^ ^,^ ^18^ ^,^ ^47^
*In vivo*	Bacterial Injection Models	• Host–pathogen interactions • TLR pathways with its cognate microorganism • Response to resuscitation and antibiotics	• Generally, mice (e.g., C57BL/6, BALB/c) or porcine models	• Cytokine and chemokine release • Organ CFUs • Survival rates	^17^ ^,^ ^18^ ^,^ ^50^
*In vivo*	Caecal Ligation and Puncture	• Polymicrobial abdominal sepsis (multi-PRR pathways) • Host-pathogen interactions • Bacteraemia • Septic shock • Organ-specific studies and dysfunction	• Mice (C57BL/6, BALB/c, CD-1)	• Cytokine and chemokine release • Survival rates • Organ injury	^17^ ^,^ ^21^ ^,^ ^40^ ^,^ ^93^
*In vivo*	CASP/CS	• Polymicrobial peritoneal sepsis • Controlled neonatal modelling (CS) • Organ dysfunction	• Mice (e.g., Swiss albino mice)	• Cytokine and chemokine release • Survival rates	^17^ ^,^ ^22^ ^,^ ^23^
*In vitro*	LPS-based Models	• Extracellular: TLR4-mediated priming (requires secondary signal for canonical inflammasome activation) • Transfection: Non-canonical inflammasome activation (caspase-4/5) via cytosolic detection • Gram-negative bacteria inflammation studies	• THP-1 • HUVECs • BMDMs • Organ-specific cells (e.g., human vascular smooth muscle cells, murine tubular epithelial cells)	• Pro-inflammatory cytokine and chemokine release • TLR4 signalling activation • GSDMD cleavage and caspase-4/5 activation (intracellular LPS) • Pyroptotic cell death markers (intracellular LPS) • Cell viability	^14^ ^,^ ^27^ ^,^ ^28^ ^,^ ^32^ ^,^ ^39^ ^,^ ^40^
*In vitro*	PAMP Combined Models	• Innate immune priming through combined NOD and TLR receptor stimulation • Polymicrobial and Gram-positive/negative studies	• THP-1 • HUVECs • BMDMs • Organ-specific cells (e.g., human vascular smooth muscle cells)	• Pro-inflammatory cytokine and chemokine release • PRR Pathway activations	^32^ ^,^ 38, 39, 40 ^,^ ^94^
*In vitro*	DAMP Models	• Sterile innate immune activation through PRR signalling • Oxidative stress • Activation of NLRP3 inflammasome post-priming (some DAMPs)	• THP-1 • HUVECs • BMDMs • Murine peritoneal macrophages	• Pro-inflammatory cytokine and chemokine release • Pathway activations (e.g., MAPK, NF-kB) • ROS/NO generation • Caspase-1 activation (if NLRP3 inflammasome is activated)	^32^ ^,^ ^42^
*In vitro*	PAMP + DAMP Combined Models	• Activation of canonical NLRP3 inflammasome pathway (caspase-1) with priming + second stimulus (e.g., ATP)	• THP-1 • HUVECs • BMDMs • Organ-specific cells (e.g., human vascular smooth muscle cells)	• Pro-inflammatory cytokine and chemokine release • PRR Pathway activations • Pyroptotic cell death	^32^ ^,^ ^39^ ^,^ ^40^ ^,^ ^95^
*In vitro*	Co-culture Models	• Cell–cell interaction (e.g., immune/endothelial, tissue/endothelial) • Immunosuppression mechanisms • Oxidative stress • Disease progression	• HUVECs and monocytes • Murine macrophages and murine T cells	• Cytokine and chemokine release • ROS/NO generation • Immunosuppression markers • Immune exhaustion • Cell Viability	^96^
*In vitro*	3D Cell Culture Models	• Cell–cell interactions (co-cultures) • Cell-ECM interactions • Gene expression fidelity	• HUVECs + macrophages (Cells in hydrogels or spheroids)	• Cytokine and chemokine release • Gene expression analysis • Cell morphology viability • Barrier integrity • 3D-specific markers	^97^
*In vitro*	Organoids	• Organ-specific injury and immune responses • Organ-specific disease development • Cell–cell interactions (co-cultures) • Cell-ECM interactions • Gene expression fidelity • Controllable cell proliferation rate	• iPSCs • Tissue-specific stem cells (e.g., liver, lung, gut)	• Cytokine and chemokine release • Gene expression analysis • Cell morphology and viability • Tissue-specific pathway activations • Organ injury • Barrier integrity • Organ-specific stress markers	^47^ ^,^ ^48^ ^,^ ^97^
*Ex vivo*	Whole Blood Stimulation	• Human immune response profiling • PRR-induced gene expression	• Human healthy donor blood	• Cytokine and chemokine release • ROS release • Cell count • Electrolyte concentrations • Humoural inflammatory activity assessment • Profiling of activation of markers of immune cells • Phagocytic activity	^56^ ^,^ ^98^ ^,^ ^99^
*In silico*	Data Mining and Machine Leaning	• Identification of molecular patterns • Prediction of therapeutic response	• Publicly available datasets	• Prediction of cytokine and chemokine gene expression • Inference of pathway activation profiles • Identification of transcriptomic markers of immune exhaustion • Prediction of survival outcomes	^64^
*In vitro*	Microfluidic Devices	• Organ-specific analysis • Polymorphonuclear leucocyte (PMN) transmigration • Real-time monitoring of immune–endothelial interactions, barrier disruption, and vascular dysfunction • Multi-organ interactions • Cell–cell interactions (co-cultures)	• HUVECs • ECs + Organ specific cells (e.g., brain endothelial like-cells with brain pericyte-like cells)	• Live monitoring of: cytokine and chemokine release, barrier function and vascular injury markers • Immune cell transmigration tracking • Dynamic quantification of endothelial dysfunction	^15^ ^,^ ^100^

This table presents a detailed overview of commonly used *in vitro*, *in vivo*, *in silico*, and *ex vivo* models in sepsis research. Each model is characterised based on experimental platform, with descriptions on their respective core focus, examples of cell types or biological samples used, and their representative measurable outcomes. The aim is to illustrate how each platform captures distinct facts of sepsis, while also highlighting to what extent each model reflects relevant features of human sepsis.

### Bench-to-Bedside gaps in sepsis models

One of the main challenges in sepsis research is translating preclinical findings into effective therapies.^7^^,^^23^ Unfortunately, many therapies fail to produce favourable results in human clinical trials, due to oversimplification of *in vitro* systems or discrepancies in animal models. Although animal models of disease may appear phenotypically close to humans, their molecular and cellular mechanisms are often different, lacking clinical relevance to humans, and potentially producing non-accurate results.^20^^,^^72^

Drotrecogin alpha activated (DAA), named Xigris, was the first biologic agent approved for sepsis based on recombinant human protein C. It was FDA-approved in 2001 and approved in the EU in 2002 based on PROWESS trial results. However, in 2011, its manufacturer, Eli Lilly and Company, announced market withdrawal after failing to benefit patients with severe sepsis and septic shock observed in the ADDRESS trial.^101^^,^^102^ This decision, driven by conflicting trial outcomes, brought attention to the gap between preclinical findings and clinical performance, underscoring the importance of critically evaluating experimental models and its translational impacts.^103^ Evidence suggests that this translational failure was influenced by the heterogeneity of the clinical sepsis population, as Lai and Thompson note that no consistent patient subgroup demonstrated clear benefit from activated protein C.^57^

Beyond broad classifications of *in vitro*, *in vivo, ex vivo*, and *in silico*, platforms rely on their distinct cellular types, design parameters, experimental features, and measured biomarkers to determine their translational impact (Table 1).

### Challenges in replicating human sepsis pathophysiology

Physiological relevance in sepsis research remains limited despite substantial progress. In contrast to organ-specific and localised infections, sepsis involves multiple organs and immune profiles, leading to varied clinical manifestations.^3^ Despite advances in cell–cell interactions, current models miss systemic dynamics present in humans. 3D cell cultures which include co-cultures, scaffold-based cultures, and organoids, enable cell–cell and cell–matrix interactions to model sepsis more precisely.^97^^,^^104^ Among them, organoids are advanced 3D systems that create miniaturised version of tissues from stem-cells.^104^ However, they lack vascular flow, shear stress changes, and circulating immune mediators that shape sepsis progression.^105^ These limitations also reflect the broader tension between improving physiological relevance and the need to reduce and refine animal use.

In contrast, animal *in vivo* models are essential for researching host-pathogen interactions, as well as organ-specific responses. Models such as CLP are used to study complex patterns of the disease like immune dysregulation and organ failure, which simpler *in vitro* models don't capture. Although widely used, species-specific responses and immune differences hinders relevance to human sepsis. Even with standardised design, translation to clinical trials may fail due to discrepancies between animals and humans.

Part I of the MQTiPSS, focused on Study Design and Humane Modelling, mentions the temporal mismatch between animal models and the clinical course of sepsis. Only 10% of the reviewed rodent studies exceed 14 days, while in humans, mortality increases by nearly 15% at the six-month mark, in comparison to 28-day outcomes. The absence of prolonged studies limits the investigation of sepsis progression overtime, meaning that these models typically capture acute endpoints rather than ICU management. Human-relevant models allow for prolonged observation under controlled conditions, which is usually not achievable in animal studies.^12^

### Implementing replacement, reduction, and refinement

Animal models have long supported disease research and drug development.^106^ Nonetheless, animal experimentation raised concerns about pain and suffering. The three Rs of animal research were introduced in 1959 by William Russel and Rex Burch: Replacement, Reduction, and Refinement.^107^^,^^108^

Most countries regulate legislation around animal testing through ethical justification as long as they promote significant potential benefits to humans. The EU, through the Directive 2010/63/EU on the protection of animals used for scientific purposes refers to the use of the 3Rs, setting out measures for animal protection in applied research.^109^ Regardless of these guidelines, the growing quantity of experiments revolving around animal testing hinders necessary vigilance of animal protection legislations.^9^^,^^110^

Part I of the MQTiPSS, brings attention to recurring issues that challenge ethical acceptability of animal-based sepsis research. Many studies still lack clear hypothesis and are conducted without proper sample size justification, which weakens confidence of findings. The frequent absence of supportive care like resuscitation or analgesia raises serious welfare concerns. It was recorded that in over 90% of the studies no euthanasia criteria were mentioned, and less than 10% of studies disclosed the use of analgesia. Part III of the MQTiPSS also provides information on the necessity of proper fluid resuscitation and antibiotic treatment, which most studies fail to report. These limitations reflect the need for greater care in how models are designed and ethically weighted.^12^

Ethical considerations go beyond welfare protocols, requiring reassessment of animal reliance, alongside of stronger commitment to developing viable alternatives. For example, the FDA is beginning to replace animal testing. In April 2025, they released a roadmap promoting the replacement of animal testing with validated New Approach Methodologies (NAMs) such as organ-on-chip, *in silico* modelling, and advanced *in vitro* assays.^111^, ^112^, ^113^ This approach, in collaboration with NIH and VA, promotes faster validation and adoption of human-relevant models.

### Model variability and its impact on reproducibility

Discrepancies between models arise from biological heterogeneity, variable stimuli, and differing experimental conditions. The gold standard for sepsis modelling, CLP, produces variable outcomes depending on caecal ligation length, size, number of punctured holes, and number of faeces that leak into the abdominal cavity. These variations reduce reproducibility. *In vivo*, divergent strains of mice can respond differently to treatment, or even same-strain mice, due to age, physiology, and genetics.^14^

An additional concern lies in the inconsistency of results obtained between the same models designs under seemingly identical conditions can yield divergent results. This suggests that subtle, unaccounted-for variables may still influence outcomes, hindering reproducibility and complicating the comparison of findings across studies and laboratories. Laboratory procedures, stress levels, and housing conditions may influence the animal and ultimately impact research outcomes, even while performing well-established protocols.^7^ These challenges further highlight the need for more rigorous standardisation, transparent reporting of methodology, and the inclusion of appropriate controls.

## Conclusion

Sepsis is a complex disease characterised by immune dysregulation, endothelial dysfunction, and multi-organ failure. Various experimental models have expanded our understanding of the disease and supported preclinical drug discovery. However, most therapeutic agents that have been proven successful in experiments, fail in human clinical trials. Translational relevance is an issue across platforms and delays the discovery of effective therapies, resulting in a higher mortality rate among sepsis patients. Reproducibility also remains a major hurdle, with variability in biological systems, protocols, and experimental design, especially around animal use.

The implementation of standardised validation frameworks is essential to ensure reproducibility and uphold ethical responsibility. The MQTiPSS guidelines present a major stepping stone in the development of animal-based sepsis models, providing fundamental standards that are equally valuable for evaluating novel models. OOC has gained increasing attention and may be used as a potential tool to reduce translational limitations of current disease models. As sepsis is a highly heterogeneous condition and involves multiple immune pathways, models or a combination of models must be based around the research question being explored. Ultimately, advancing sepsis modelling through human-relevant, ethical, and standardised approaches is essential to enhance disease understanding and accelerate the development of biomarkers and therapeutic solutions. This should be done in alignment with the MQTiPSS as a guide to ensure that the new model is held to a high level of scientific and ethical integrity.

### Outstanding questions

Progress in sepsis research has been moulded by attempts to replicate isolated mechanisms of the disease, such as immune dysfunction, organ failure, or inflammatory cascades. While each model has contributed to the field, their fragmented nature has perpetuated translational limitations. Current challenges include the absence of standardised experimental criteria, limited comparability between animal and human responses, and ethical constraint surrounding animal experimentation. Model choice should be guided by the specific mechanisms under investigation, while ensuring reproducibility and alignment with recognised standards.

Looking ahead, rather than solely refining existing models, future research should move towards integrating complementary systems capable of capturing multiple disease dimensions, ultimately adding value to established knowledge. Microfluidic devices have shown considerable success in studying lung, liver, kidney, heart, skin, and brain tissue, increasing knowledge in fields such as cancer and immunology.^114^ These are valuable tools for real-time sepsis research, capable of addressing complex disease dynamics not yet explored in other models. For example, organ-on-chip can enable real-time study of endothelial permeability, leucocyte adhesion, and microvascular leakage. Microfluidic models used alongside treated animal studies may link human-cell mechanisms to systemic physiology, strengthening translation.

This strategy also reduces animal use by providing a human-like response without animal experimentation. Adopting these systems, alongside *in silico* technologies, will move sepsis research towards higher precision and clinical relevance. This will depend on interdisciplinary training and investments in infrastructure, increasing collaboration between clinicians, biologists, and engineers. Regulatory agencies should lay guidelines for validating and incorporating this technology into disease model applications and therapeutic validation. Ultimately, advancing sepsis modelling through human-relevant, ethical, and standardised approaches is essential to enhance disease understanding and accelerate the development of effective therapeutic agents.

**Search strategy and selection criteria**

All data for this review was extracted through PubMed and Scopus. Relevant literature was identified using the following keywords in different combinations: “Sepsis”, “Experimental Models”, “Preclinical Models”, “*In vivo* Models”, “Animal Models”, “*In vitro* Models”, “*Ex vivo* Models”, “*In silico* Models”, “Caecal Ligation and Puncture”, “Ethical Issues”, “Organ-on-chip”, “Microfluidic Technology”, and “Microfluidics”. Only English-language peer-reviewed articles with clear methodologies were included. Additionally, reference lists of selected articles were screened to identify further relevant studies.

## Contributors

M.J.S: Conceptualisation, Writing—original draft, Writing—review and editing, Visualisation; G.W.F: Conceptualisation, Writing—original draft, Writing—review and editing, Supervision; R.P: Writing—review and editing, Supervision; P.M: Writing—review and editing, Supervision; E.R: Writing—review and editing, Supervision; J.G.F: Conceptualisation, Writing—review and editing, Supervision; E.J.M: Conceptualisation, Writing—original draft, Writing—review and editing, Project Administration, Supervision. All authors have read and approved the final version of the manuscript.

## Declaration of interests

The authors declare no competing interests.
